# Low expression of lysosome-related genes KCNE1, NPC2, and SFTPD promote cancer cell proliferation and tumor associated M2 macrophage polarization in lung adenocarcinoma

**DOI:** 10.1016/j.heliyon.2024.e27575

**Published:** 2024-03-02

**Authors:** Zi-Ming Wang, Zhi-Lin Ning, Chao Ma, Tang-Bin Liu, Bo Tao, Liang Guo

**Affiliations:** a Department of Thoracic Surgery, Shanghai Pulmonary Hospital, Tongji University School of Medicine, Shanghai 200443, China; b Key Laboratory of Computational Biology, Shanghai Institute of Nutrition and Health, University of Chinese Academy of Sciences, Chinese Academy of Sciences, Shanghai 200031, China; c Department of Thoracic Surgery, The First Affiliated Hospital of Zhengzhou University, Zhengzhou University, Zhengzhou 450052, Henan, China; d Department of Thoracic surgery, Anhui Chest Hospital, Hefei 230061, Anhui, China

**Keywords:** Lung cancer, Lysosome, Prognosis, Immunotherapy, Macrophage

## Abstract

**Background:**

Recent research has shown that lysosomes play a critical role in the onset and progression of malignancy by regulating tumor cell death through several mechanisms. Nevertheless, the involvement of lysosome-associated genes (LSAGs) in lung adenocarcinoma (LUAD) is still not well understood.

**Methods:**

LSAGs were identified in malignant lung epithelial cells, as well as biologically and functionally annotated by the comprehensive integration of single-cell and bulk RNA-sequencing data. Prognostic characterization of LSAGs was established, of which the accuracy and reliability were assessed by one-way Cox and LASSO regression. Correlations between LSAG properties and immune cell infiltration, chemotherapy, and immunotherapy were analyzed by integrated omics data. Finally, we characterized the expression of three LSAGs (*KCNE1*, *NPC2*, and *SFTPD*) in malignant lung epithelium and assessed their impact on tumor malignancy related phenotypes.

**Results:**

We identified 18 LSAGs associated with prognosis, of which 3 LSAGs were used to construct prognostic models. High-risk patients had worse survival and the model predicted it better than other clinical indicators. Based on the functional enrichment analyses, LSAGs were associated with binding and molecular activity functions, inhibition of DNA damage repair and tumor growth, IL7 signaling pathway, and glycolysis. M0 macrophages and M1 macrophages were substantially enriched in high-risk patients. Conversely, there was a considerable enrichment of resting dendritic cells and M2 macrophages in patients at low risk. We also found that risk scores predicted the outcome of immunotherapy. In vitro, we found that KCNE1, NPC2, and SFTPD were lowly expressed in malignant epithelial cells and patients with low expression of KCNE1, NPC2, and SFTPD had a higher percentage of M2 macrophage infiltration. Overexpression of KCNE1, NPC2, and SFTPD suppressed the proliferation and invasion of malignant cells, and M0 macrophages remarkably reduced M2 macrophage polarization and cellular secretion of pro-tumor cytokines.

**Conclusions:**

We used three LASGs—KCNE1, NPC2, and SFTPD—to develop and validate a predictive signature for LUAD patients. Furthermore, we found that low expression of KCNE1, NPC2, and SFTPD promotes lung cancer cell proliferation and invasion and M2 macrophage polarization. Our study may provide fresh perspectives for customized immunotherapy.

## 1 Introduction

Globally, lung cancer (LC) is the one of the most prevalent carcinomas [1]. With over a million fatalities annually, it becomes a leading contributor from cancer to death, which is also linked to a dismal prognosis [2]. In clinical, it is reported that over 40% of lung cancer instances are lung adenocarcinoma (LUAD), making it as the most prevailing subtype of this disease [3]. The recurrence rate of LUAD is still significant, even though the knowledge of its molecular foundation, diagnosis, and therapy has made enormous strides [4]. Despite significant advancements in targeted treatment and chemotherapy for lung cancer, the overall survival (OS) rate for the majority of patients remains dismal [5]. A major contributing factor is that the majority of patients get their diagnoses after the disease has been progressed to an advanced stage [6]. Currently, there were several commonly used clinical predictors of LUAD prognosis including tumor size, metastasis, and mutational load [7]. However, in practice, the specificity of those clinical properties is sub-optimal for prognosis prediction because if the heterogeneity of the tumors, i.e. patients with a similar tumor-node-metastasis (TNM) stage greatly differs in the treatment outcome and prognosis [8]. Therefore, it is necessary to investigate potential biomarkers, which could be complementary with clinical predictors, to help treatment and prognostic prediction and facilitate the development of LUAD personalized diagnosis and therapy.

Lysosomes are single-membrane vesicle structures that contain a multitude of acidic hydrolytic enzymes with an internal pH of approximately 4.5–5.5, which stimulates the production of macromolecular precursors [9,10]. Endocytosis-engulfed defective proteins and organelles can be degraded by lysosomes via the release of acidic hydrolases, while cells reabsorb nutrients in the form of metabolites to preserve metabolic balance, which is considered as the “garbage disposal system” and the “suicide bag” of cells [[11], [12], [13]]. Lysosomes are also widely acknowledged as the cellular and organismal homeostasis regulator [14]. It plays an important role in signal transmission, metabolic adaptability, cellular proliferation and differentiation, cell secretions, and quality control of organelles and proteins.

The lysosomal-induced cell death is called as lysosomal cell death, which was critical for the mechanism exploration for cancer cells proliferation. A systematic investigation of the function of lysosomes in LUAD has not yet been conducted. Lysosomal enzyme activities are comparatively diminished in surrounding normal tissues in comparison to malignant tissues [15]. Lysosomal dysfunction and function influence a range of cancer biological markers. Hence, it is essential to comprehend the lysosomal alterations that occur in cancer to develop medicines that specifically target this organelle [16]. At present, immunotherapy is emerging as a prominent therapeutic approach for lung cancer. Our curiosity was piqued by previous research in this domain to investigate the prognostic and predictive significance of LSAGs, as well as their relevance to anti-tumor immunity in LUAD [17]. Therefore, understanding the factors influencing lysosomes and how to regulate lysosomes will be useful in cancer diagnosis and prognostic prediction. Nevertheless, the involvement of LSAGs in carcinogenesis and progression has only been revealed in few studies.

This study established a new clinical risk model for LSAGs to predict prognosis and immunotherapy efficacy in lung cancer. We used a prognostic model to evaluate the prognosis, clinical relevance, and immunotherapy in the TCGA group and performed external validation in the GEO database. These findings suggest that novel multi-molecular diagnostic models based on LSAGs can evaluate the prognosis of LUAD. Additionally, after studying LSAGs, we found that the expression levels of KCNE1, NPC2, and SFTPD were low in malignant epithelial, and the proliferation and invasion capacity of cells was inhibited by the over-expression of these genes. Further studies revealed that M2 macrophage infiltration level was higher in patients with reduced KCNE1, NPC2, and SFTPD expression. Therefore, to further co-culture H1299 cells with HCC827 and M0 macrophages, we observed that after the over-expression of KCNE1, NPC2, and SFTPD, the M2 polarization of M0 macrophages was significantly reduced, and the content of tumor-promoting cytokines secreted by the cells was significantly reduced. This study provides a theoretical framework for anti-tumor strategies targeting LSAGs.

## 2 Materials and methods

### 2.1 Data collection

The 585 LUAD patients were collected from the TCGA database. Clinical and pathological data was collected, including information on gender, age, TNM classification, survival status, tumor staging, and survival outcomes. Among these participants, 328 patients were alive, and 186 patients were deceased; 223 patients aged ≤65 years and 273 patients aged >65 years; 275 females and 239 males. Pathological staging revealed that there were 385 cases in stages I and II, 106 cases in stages III and IV, and 6 cases with unknown staging. For T staging, there were 432 cases with T0, T1, or T2, 54 cases with T3 or T4, and 11 cases with unknown stage. Regarding the metastasis (M) status, there were 317 cases with M0, 25 cases with M1, and 150 cases with unknown status. For lymph node (N) staging, there were 312 cases with N0, 168 cases with N1, N2, or N3, and 17 cases with unknown stages (see Supplementary Table S1). The single-cell dataset was downloaded from the GEO website with the accession number GSE117570. The lysosome-related gene set is derived from the msigdb database, comprising a total of 61 genes.

### 2.2 Single-cell data quality control

The LUAD single-cell discovery dataset, generated using the cellranger package, was used to create a Seurat object with gene expression matrices and sample annotation information. Subsequent single-cell analysis was conducted using Seurat v3.1.4. In the quality control step, cells with detected gene counts between 100 and 6000, UMI counts greater than 200, and lysosomal gene expression less than 10% were retained. Standard Seurat procedures were followed, including normalization, identification of highly variable genes, scaling, principal component analysis, and batch effect correction using Harmony. Cells with cumulative variance reaching 80% were retained for further clustering.

### 2.3 Clustering and cell annotation

Clustering was performed using the optimal resolution value for t-SNE visualization. For improved cell annotation accuracy, tumor and normal tissue-derived samples were not distinguished at this stage. Cell sub-types were annotated based on molecular expression patterns. The FindAllMarkers function in Seurat was employed to explore deferentially expressed genes between sub-types and groups, using the Wilcox test as the statistical method with default parameters.

### 2.4 Single-cell trajectory analysis

Monocle2 was used to explore the evolutionary processes among different macrophage sub-types. Monocle2 is a key tool for pseudo-time analysis of single-cell transcription data, simulating cell developmental trajectories or sub-type evolution based on unsupervised learning (reversed graph embedding algorithm). Using the expression matrix of individual cells, Monocle2 first extracts the expression patterns of key genes and then ranks individual cells in pseudotime, ultimately simulating the differentiation trajectory or sub-type evolution of cells during the developmental process. Monocle2 typically generates a tree-like structure for cell trajectories, with one end as the “root,” representing the starting state of the biological process, and the other end as the “leaf” where cells are arranged based on their pseudotime values, indicating their position along the trajectory. Finally, functions such as plot_cell_trajectory and plot_genes_in_pseudotime were used to visualize the cell trajectory.

### 2.5 Single-cell copy number analysis

CopyKAT (Copy number detection from Karyotyping Analysis Toolkit) is used for detecting chromosomal copy number variations (CNV) from single-cell RNA sequencing data. Its main goal is to reveal heterogeneity by analyzing copy number variations in individual cells. We identified normal and malignant cells based on copy number changes. The FindMarkers function was employed to perform differential analysis between the two heterogeneous cell types.

### 2.6 Construction of a clinical prognostic model

First, the intersection of deferential expressed genes (DEGs) and lysosome-related genes was determined. Then, the R caret package was applied to perform group cycling on the gene expression matrix with complete clinical information. The dataset was classified at random into a training set (train) and a test set (test) in a 7:3 ratio. The training set was chosen to build a lung cancer risk prognosis model, and the test set was used to evaluate the performance of the risk prognosis model. We employed a 10-fold cross-validation, dividing the dataset into 10 subsets. During the training process, the model was trained on 9 subsets, and then its performance was evaluated on the remaining subset. This process was repeated 10 times, ensuring that each subset was used for validation once. The risk estimates for each of the 585 lung cancer samples in the TCGA database were calculated using the lysosome-related gene prognostic model. The next step involved dividing patients into high-risk (≥ median risk value) and low-risk (< median risk value) subgroups. Then, the Kaplan-Meier method was employed to evaluate patients' survival across the two subgroups and plot the curves. The Receiver Operating Characteristic (ROC) curve was generated for the established model, the area under the curve (AUC) was computed, and the prediction accuracy was interpreted. The rms and survival packages were utilized to construct nomogram predicting the 1-, 3-, and 5-year survival rates.

### 2.7 Immune infiltration analysis

Different immune cells play different roles. We employed the R CiberSoft program to analyze the proportions of immune cells in lung cancer patient samples to precisely determine the immunological composition in the tumor microenvironment (TME). The input files consist of expression data and a leukocyte feature matrix file (LM22.txt). We utilized the R ggpubr package to visualize the statistical test results after comparing the differences in immune cell proportions between high-risk and low-risk patients using the Wilcoxon rank-sum test. The tumor microenvironment (TME), which comprises tumor cells, stromal cells, and immune cells, was assessed for lung cancer patients using the ESTIMATE algorithm. For this study, we analyzed the correlation between the TME and risk scores utilizing the ggpubr and stats packages.

### 2.8 Immunotherapy response and drug sensitivity prediction

The IMvigor210 cohort was employed to evaluate the effectiveness of anti-PD-L1 immune therapy, specifically predicting how high and low-risk patients respond to Immune Checkpoint Blockade (ICB) treatment. Using the chi-square test, we assessed the response differences to immunotherapy among patients with different risk scores. Leveraging the drug genomics database (GDSC), we conducted analyses using the R package “oncoPredict” to predict the drug sensitivity for each sample. Simultaneously, we estimated the IC50 values for each sample and employed the Wilcoxon rank-sum test to statistically evaluate the variations in drug sensitivity between high- and low-risk patients.

### 2.9 Cell culture and treatment

BEAS-2B cells were purchased from ATCC (RRID: CVCL _ 0168), lung cancer cell line H1299 (RRID: CVCL _ 0060), A549 (RRID: CVCL _ 0023) and HCC827 (RRID: CVCL _ 2063) cells were obtained from CCTCC. A DMEM medium that contained 10% FBS (fetal bovine serum) was used for cell culture and the medium was replaced until the cell concentration reached 85%. Cells from passages 3 to 10 were used for the experiments. All cell lines were verified by STR.

### 2.10 qRT-PCR

The Trizol reagent was employed to isolate total RNA from the cells. Following that, the PrimeScript RT Reagent Kit was utilized to conduct reverse transcription. Then, quantitative real-time PCR amplification was conducted on an Applied Biosystems 7500 real-time PCR system following the protocols of the SYBR Premix Ex *Taq*II Kit.

### 2.11 Western blotting (WB)

Protein samples were obtained by using RIPA lysates containing protease inhibitors. Utilizing the Double (BCA) Protein Quantification Kit's guidelines (BCA 1-1 KT; Sigma Aldrich Chemical Company), the protein concentration of every sample was quantified. After that, a 10% sodium dodecyl sulfate (SDS) polyacrylamide gel was used to separate the proteins via electrophoresis, after which, the protein was moved from the glue to a nitrocellulose membrane with a pore diameter of 0.2 μmol/L. In addition, to block non-specific binding sites, a solution of 1 tris buffer salt Tween (TBST) with 5% skim dry milk was added to the membrane, and it was gently shaken for 1 h at room temperature (RT). The membrane was subjected to incubation with the primary antibody and then incubated overnight at 4 C with GAPDH as an internal reference. Secondary antibodies were introduced, and the reaction was performed for 1 h at RT. In addition, an electrochemiluminescence (ECL) reagent (Sigma Aldrich Chemical Company) was employed to visualize the protein bands. The ratio of target and inner reference bands represents protein levels as determined by Image Pro Plus 6.0 (Media controlnetics, San Diego, California), and the internal reference used was GAPDH.

### 2.12 CCK-8

First, 96-well plates were seeded with logarithmic-phase LC cells (100 μ L) (1 × 10^4^ cells). After 24 h of incubation (37 °C, 5% CO2), the medium was replaced and processed. Then, for 1, 2, 3, 4, or 5 days, the cells were treated with CCK-8. With a Bio-Rad instrument, we measured the absorbance at 450 nm and averaged the results from 5 wells for every treatment.

### 2.13 EdU

The EdU-Apollo 567 in vitro kit (Ribbio, Guangzhou, China) was utilized to examine cell proliferation. A 96-well plate was used for cell culture of the transfected cells (1 × 10^4^/well). EdU-labeled medium (50 μM) was introduced to the cells after 48 h, and they were subjected to incubation for another 2 h. We used a combination of 4% paraformaldehyde (PFA) to fix the cells, 0.5% Triton X-100 in PBS to permeabilize them, then Apollo staining solution and DAPI to stain them. We used an inverted fluorescence microscope (IX73-AIZFL/PH) (Olympus, Japan) to capture images of the cells.

### 2.14 Transwell

First, 50 μ L of Matrigel gel (BD Bioscience, USA) was applied evenly on the upper surface of the transwell (8- μ m aperture; Corning Costar, Cambridge, MA, USA) and then gently placed in the wells of a 24-well plate and incubated overnight to cure the gel. After trypsin digestion, a serum-free DMEM medium was used to resuspend LC cells, and the density of the cells was adjusted to 2105 cells/ml. The bottom chamber was loaded with 250 μL DMEM medium and 10% FBS, 100 μ LLC cell suspension was introduced into the top chamber, and the plates were subjected to incubation for 24h. Thereafter, inserts were collected and cells uninvaded or unmigrated upper surface were removed with cotton swabs, and cells invading the bottom wells were directly fixed in 4% PFA for 15 min and subsequently captured with crystal violet for 15 min on a fluorescence microscope (IX-71, Olympus, Japan). The count of cells in the bottom wells was determined with Image J software (NIH, Bethesda, MD, USA).

### 2.15 Induction by M0 macrophages

RPMI-1640 medium that contained 10% FBS and 1% penicillin/streptomycin was utilized to culture THP-1 cells (2–5*10^5^ cells/mL). For 48 h, cells were exposed to 100 ng/mL of 12-myristate 13-ethyl ester (PMA) to induce M0 macrophage differentiation. After PMA treatment, cells were washed with a new medium, and incubation in a new medium was continued for 24 h to promote further differentiation of M0 macrophages.

### 2.16 Co-culture

The upper surface of the transwell membrane was coated with Matrigel and lung cancer cells were seeded into the upper compartment of the transfer system at 1–2*10^5^ cells/mL using RPMI-1640 medium containing 10% FBS and 1% penicillin/streptomycin. M0 macrophages were seeded into the lower compartment of the transfer system at 1–2*10^5^ cells/mL using RPMI-1640 medium with 10% FBS and 1% penicillin/streptomycin. The transfer system was incubated at 37 °C in the 5% CO_2_ moist atmosphere for 72 h, and subsequently, the cytokines secreted by M2 macrophages by ELISA and the expression of the M2 cell marker Arg 1 by immunofluorescence of macrophages by immunofluorescence.

### 2.17 Immunofluorescence

In 24-well plates, co-cultured macrophages were seeded and allowed to grow until they reached 50% confluence. Following a two-step PBS washing, 4% PFA was used to fix the samples for 15 min at RT, and 0.5% Triton X-100 was used to permeabilize them for 2min at RT. This was followed by overnight incubation with anti-Arg 1 antibody (1:200) at 4 °C, 1h with FITC bound secondary antibody, and counterstaining with DAPI for 10 min at RT. A confocal fluorescence microscope (Nikon Corporation, Tokyo, Japan) was used to capture images of the stained cells.

## 3 ELISA

The cell supernatant was chilled at 4 °C for half an hour and centrifuged at 3000 r/min. The concentration of inflammatory factors in the joint synovial fluid was determined using IL-10, IL 13, and the TGF- β ELISA kit (Elabscience, Wuhan, China). Then, 100 μL standard working solution or samples was introduced into each well and incubated at 37 °C for 90 min. Then, a 100 μL working solution that was biotinylated with anti-IL-I0, IL-13, and TGF- β was introduced and incubated at 37 °C for 1 h. Finally, a 100 μL working solution that was conjugated with HRP was introduced and incubated at 37 °C for half an hour. A 90 μL substrate solution was then introduced and subjected to incubation at 37 °C for approximately 15 min. The next step was to add 50 μL of the terminator solution. The readings were taken immediately at 450 nm, using an iMark Microplate reader (Bio-Rad, USA).

### 3.1 Data analysis

SPSS22.0 (IBM SPSS Statistics, Chicago, IL, USA) was utilized to analyze statistical data. The format of mean ± SD was used to present the measurement data. The unpaired *t*-test was employed to assess differences between the two groups. Data between multiple groups were compared using one-way ANOVA or Two-Way ANOVA, Tukey's post hoc test, and post-statistical analysis using log rank test indicated statistical significance at *P* < 0.05.

## 4 Results

### 4.1 Single-cell data pre-processing

**Fig. 1:**
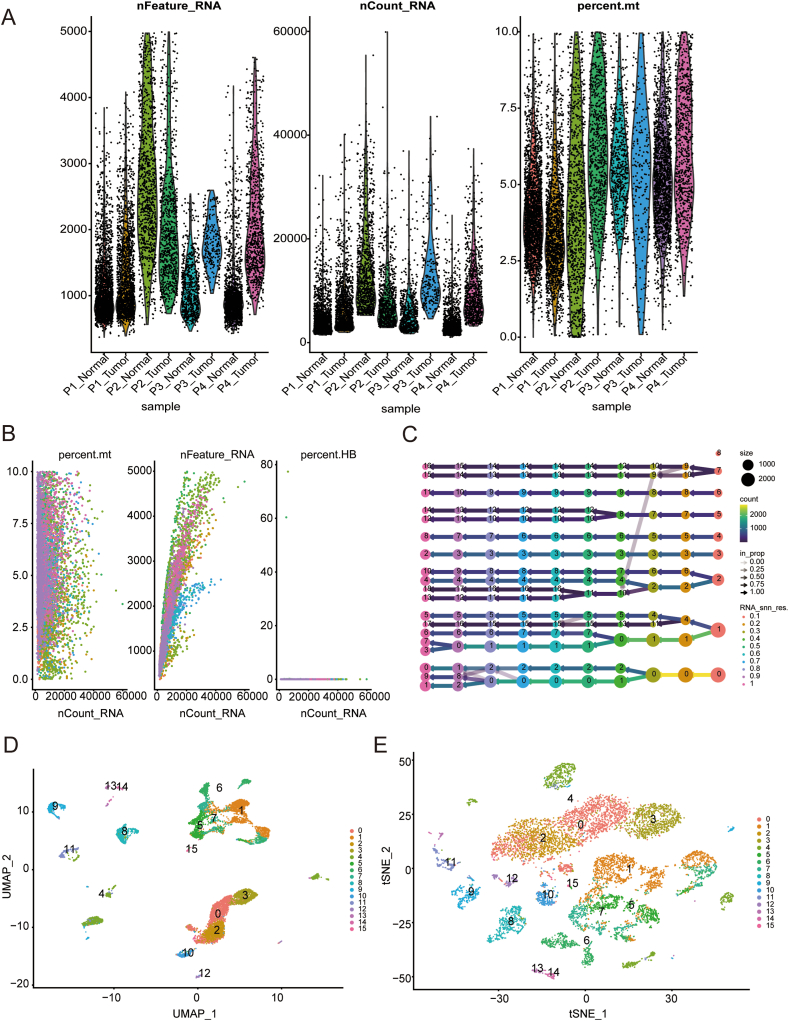
Preprocessing and clustering analysis of GSE117570 single-cell data. (A): Illustration of the quality control process, resulting in the retention of 60,288 cells. (B): Correlation analysis depicts the relationship between RNA count, features count, and red blood cell count. (C): Optimal clustering occurred at a resolution of 0.5 for accurate cell grouping. (D–E): Clustering results were visualized using both umap and t-SNE dimensionality reduction methods. (For interpretation of the references to colour in this figure legend, the reader is referred to the Web version of this article.)

Firstly, we filtered the dataset based on the gene counts and the percentage of lysosomal genes. From the quality control plot, it can be observed that we retained 60,288 cells by controlling for the number of detected genes (>200 and < 8000) and the percentage of lysosomal gene expression (<10%) (Fig. 1A). Further analysis revealed the correlation between RNA count, lysosomal content, and red blood cell count (Fig. 1B). For accurate clustering, we observed the clustering results with resolutions ranging from 0.1 to 1 and found that the optimal clustering occurred at a resolution of 0.5 (Fig. 1C). Finally, we visualized the clustering results using both umap and t-SNE dimensionality reduction methods (Fig. 1D–E).

### 4.2 Cell annotation

**Fig. 2:**
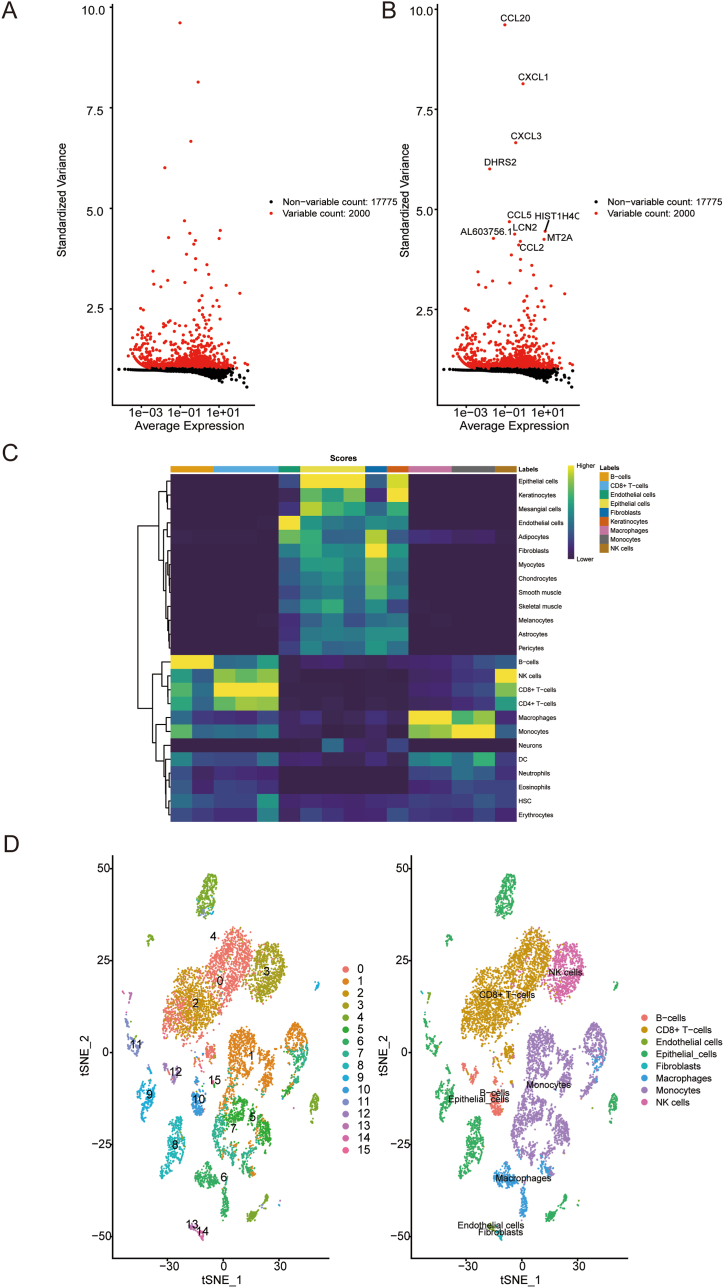
Cell Annotation Using the singleR Package. (A–B): Top 3000 genes capturing the overall variation in the dataset based on standard deviation. (C): Cell scores across all reference labels, indicating confidence in predicting labels throughout the single datasets. (D): Distribution of the annotated cell types (epithelial cells, fibroblasts, endothelial cells, macrophages, CD8^+^ T cells, monocytes, B cells, and NK cells) is visualized using t-SNE dimensionality reduction.

Next, we utilized the singleR package for cell annotation. Initially, we identified highly variable genes, which measure the degree of variation between cells based on standard deviation. The variation of the top 3000 genes was found to represent the overall variation information in the dataset (Fig. 2A). We then used the plotScoreHeatmap function to display the scores of all cells in all reference labels, checking the confidence of predicting labels throughout the dataset, and showing ideal annotation results (Fig. 2B). We annotated a total of 8 cell types: fibroblasts, endothelial cells, epithelial cells, CD8^+^ T cells, macrophages, B cells, monocytes, and NK cells. Additionally, we visualized the distribution of cell types based on t-SNE dimensionality reduction (Fig. 2C and D).

### 4.3 Pseudotime analysis

**Fig. 3:**
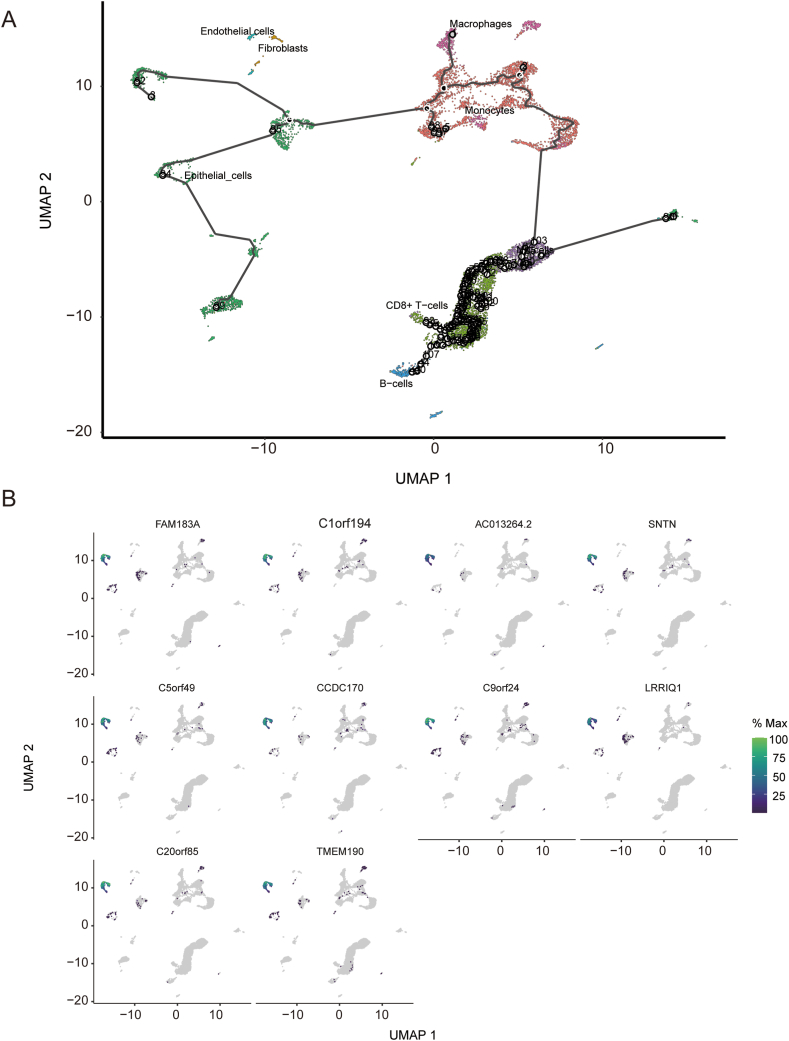
Pseudotime Analysis Using DDRTree for Cell Sorting. (A): The graph represents dimensionality reduction using the DDRTree method, with the x and y axes indicating two principal components. Each dot corresponds to a cell, and black circles with accompanying numbers denote nodes determining distinct cell states in the trajectory analysis. (B): Coordinates of the trajectory time-related expressed genes are displayed, providing insights into the gene expression patterns along the pseudotime trajectory.

We employed the DDRTree method for dimensionality reduction and further cell sorting based on pseudotime analysis. The x and y axes represent two principal components, with each dot representing a cell. The black circles in the graph with accompanying numbers represent nodes determining different cell states in the trajectory analysis. It is observed that epithelial cells have multiple differentiation trajectory nodes, indicating transcriptional heterogeneity (Fig. 3A). We also displayed the coordinates of the trajectory time-related expressed genes (Fig. 3B).

### 4.4 Single-cell copy number analysis

**Fig. 4:**
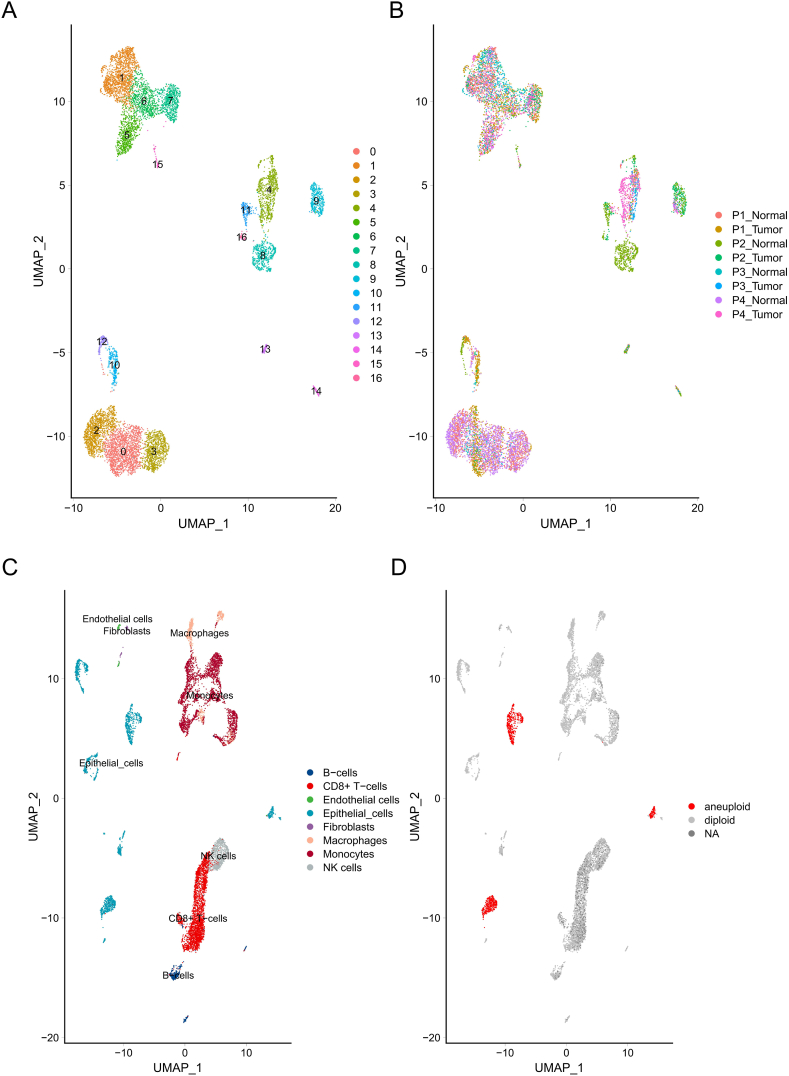
CNV Analysis and Cell Classification. (A–B): The distribution of single-cell samples is plotted. (C–D): The CNV analysis results are overlaid with the UMAP clustering results. Identified malignant cells are highlighted, and it is observed that these cells are predominantly epithelial cells. This indicates the coexistence of normal and malignant states within the epithelial cell population.

We calculated the CNV signal for all cells in the dataset. Cells with a mean square variance greater than 0.02 or consistency with the CNV signal of the top 5% of cells with the highest mean square variance greater than 0.2 were considered malignant cells, while others were considered normal cells. Firstly, we plotted the distribution of single-cell samples (Fig. 4A and B). The CNV analysis results were overlaid with the UMAP clustering results (Fig. 4C and D). It was found that the identified malignant cells were mainly epithelial cells, indicating the coexistence of malignant and normal states in epithelial cells.

### 4.5 Differential expression analysis of normal and malignant epithelial cells

**Fig. 5:**
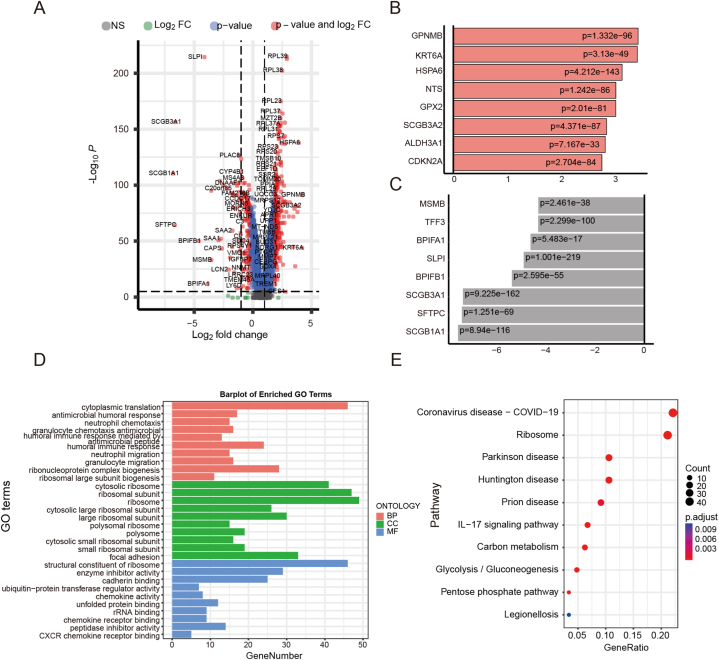
Differential expression analysis of normal and malignant epithelial cells. (A): The volcano plot illustrates the results of the differential analysis between normal and malignant epithelial cells, identifying a total of 3290 differentially expressed genes. (B–C): Bar plots display the top 8 upregulated and downregulated genes among the identified differentially expressed genes. (D): GO analysis illustrated the association of differentially expressed genes with binding and molecular activity functions, known to inhibit DNA damage repair and tumor growth. (E): KEGG enrichment analysis identifies signaling pathways, including the IL7 signaling pathway and glycolysis, enriched between normal and malignant epithelial cells.

Subsequently, we conducted differential analysis between malignant and normal epithelial cells and obtained a total of 3290 DEGs, we employed the Wilcoxon rank-sum test to calculate the significance of differential expression for each gene, and applied multiple testing correction to reduce the false positive rate. The filtering threshold was set as padj <0.05 and |log2FC| > 1 (Fig. 5A). We also displayed the top 8 upregulated and downregulated genes (Fig. 5B and C). To evaluate the underlying molecular mechanisms of these DEGs, we performed GO analysis. The results revealed that differentially expressed genes are associated with binding and molecular activity functions, known to inhibit DNA damage repair and tumor growth (Fig. 5D). KEGG enrichment analysis identified signaling pathways, including IL7 signaling pathway and glycolysis, enriched between low-risk and high-risk patients.

### 4.6 Constructing a prognostic model of deferentially expressed LSAGs

**Fig. 6:**
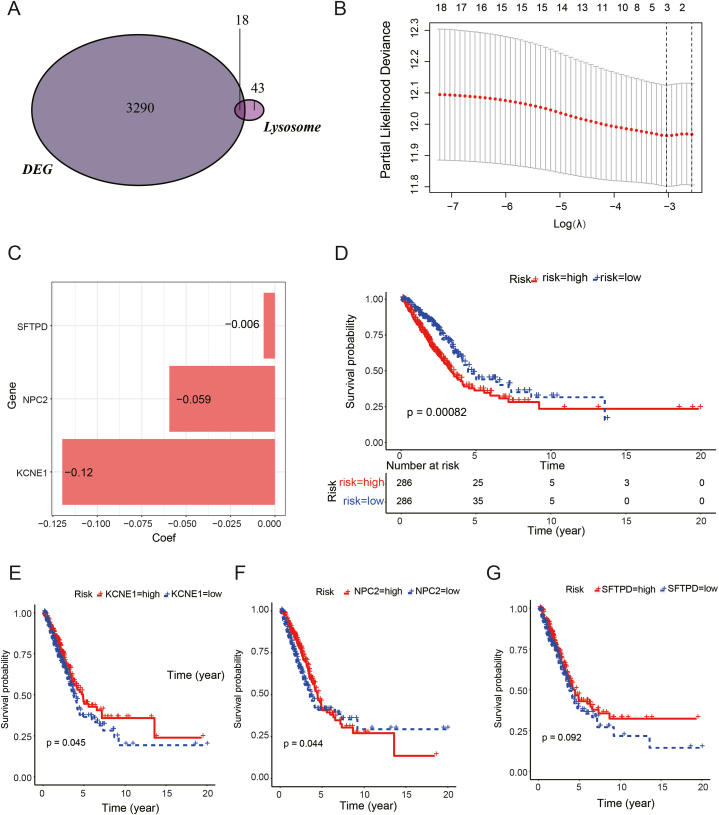
Lysosomal-Related Genes and Risk Model Construction. (A): The Venn diagram displays the 18 genes associated with lysosomes obtained from the intersection of differentially expressed and lysosomal-related genes. (B–C): Three genes—SFTPD, NPC2, and KCNE1—were selected for inclusion in the risk model by LASSO Cox regression analysis conducted in the TCGA database. (D): The Kaplan-Meier survival curve indicates significantly lower overall survival (OS) for high-risk patients compared to those at low risk. (E–G): Survival analysis of the three genes (SFTPD, NPC2, and KCNE1) revealed them to be protective factors.

**Fig. 7:**
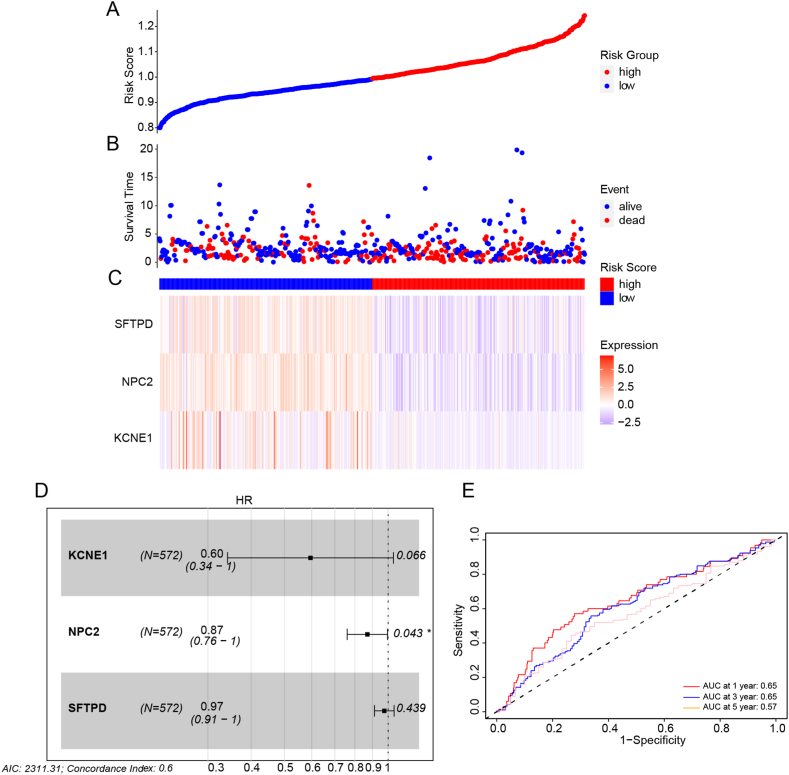
Risk model evaluation. (A–B): The scatter plot illustrates the distribution of risk scores and the corresponding survival status of lung cancer patients. (C): The expression of SFTPD, NPC2, and KCNE1 genes in low- and high-risk patients. (D): The forest plot displays hazard ratio (HR) values for SFTPD, NPC2, and KCNE1, with all values less than 1 and NPC2 reaching statistical significance. (E): The risk model's excellent predictive power is shown by the ROC curves for 1,3, and 5-year survival.

The intersection of DEGs and lysosomal-related genes yielded 18 genes associated with lysosomes (Fig. 6A). Through LASSO Cox regression analysis in the TCGA database, three genes (SFTPD, NPC2, and KCNE1) were identified to establish a risk model (Fig. 6B). Risk scores were calculated using the coefficients of these genes based on the formula below: Risk Score = SFTPD × (−0.006) + NPC2 × (−0.059) + KCNE1 × (−0.2) (Fig. 6C). Each patient's risk scores that were calculated using the risk score formula. Lung cancer patients were then stratified into high- and low-risk patients as per the median risk score(table S1). As shown in Fig. 6D, patients with higher risk had a considerably poorer overall survival (OS) than those at lower risk, according to the Kaplan-Meier survival curve analysis (*P* = 0.00082). Additionally, the three genes were shown to be protective factors through survival analysis (Fig. 6E–G). Fig. 7A displays the distribution of risk scores and the survival status. The forest plot indicates that HR values for SFTPD, NPC2, and KCNE1 are all less than 1 (Fig. 7B), with NPC2 reaching statistical significance. Subsequently, ROC curves for 1, 3, and 5 years were generated, demonstrating the strong predictive capabilities of the model (Fig. 7C). Based on these findings, the risk model serves as a crucial indicator for assessing the prognosis of lung cancer patients.

### 4.7 Correlation between prognostic model and clinical indicators

**Fig. 8:**
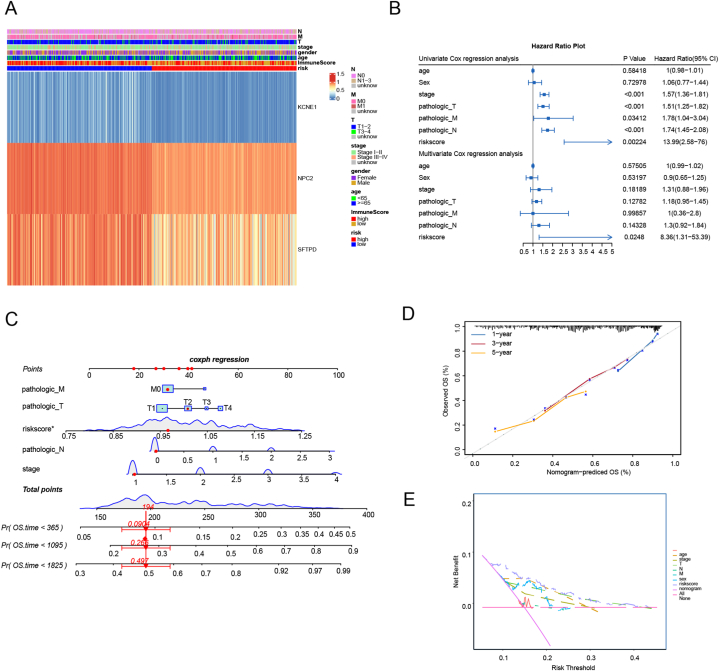
Correlation between prognostic model and clinical indicators. (A): Subgroup analysis revealed significantly higher overall survival rates for low-risk patients compared to those at high risk in different age groups, genders, races, T/N/M stages, and tumor stages (p < 0.05). (B): Clinical data, including TNM staging, were subjected to single-factor and multi-factor Cox regression analyses, based on risk scores. (C): Nomogram was plotted to facilitate the clinical application of the prognostic model. (D): Standard curves were generated to assess the model's predictive efficacy. (E): DCA was conducted to compare the predictive efficacy of the model with other clinical indicators.

To assess the effectiveness of the prognostic model, patients were stratified into subgroups depending on clinical information including age, gender, clinical stage, etc. High and low-risk comparisons were made among these subgroups. Subgroup analysis revealed that low-risk patients had significantly higher overall survival rates than high-risk patients of different ages, genders, races, T/N/M stages, and tumor stages (*p* < 0.05) (Fig. 8A). In addition, clinical data, such as TNM staging, were subjected to univariate and multivariate Cox regression analyses, based on risk scores (Fig. 8B), indicating that our model can serve as an independent predictor for patient outcomes. Nomogram were plotted to facilitate the model's clinical application (C-index = 0.792) (Fig. 8C). Finally, standard curves and decision curve analysis (DCA) were conducted, showing that the model's predictive efficacy surpassed other clinical indicators (Fig. 8D–E).

### 4.8 Immune analysis of low- and high-risk patients

**Fig. 9:**
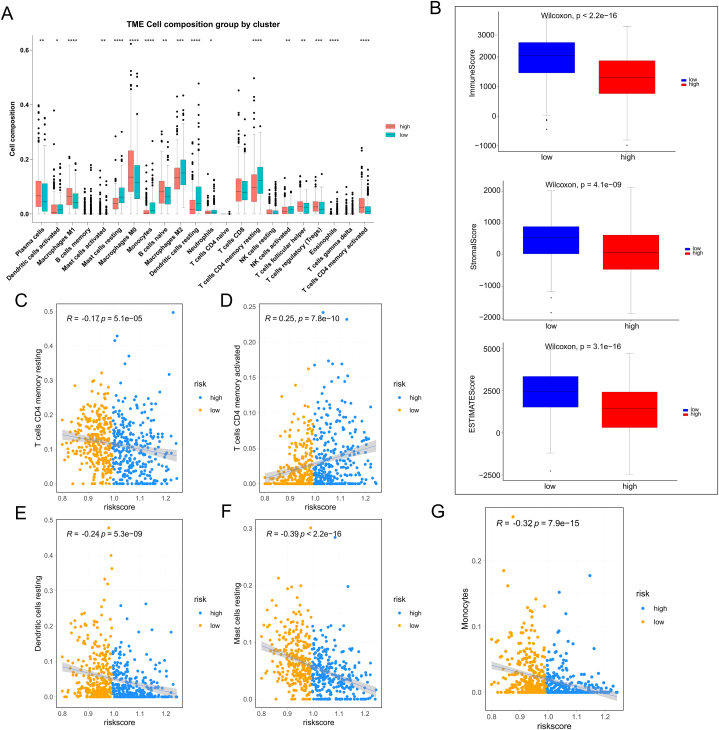
Immune Cell Analysis and Correlation with Prognostic Model. (A): Significant enrichment of M0 and M1 macrophages was observed in the high-risk patients, while M2 macrophages and resting dendritic cells were strongly enriched in the low-risk patients. (B): For both high- and low-risk patients, the stromal, immune, and ESTIMATE scores were computed using the ESTIMATE method. (C–G): Immune-infiltrating cells were examined in connection to risk scores. Risk scores were negatively related to four immune cells (resting dendritic cells, monocytes, resting mast cells, and resting CD4^+^ T cell memory) and positively related to activated CD4^+^ T cell memory.

Next, variations in 23 immune cells were compared between patients with high and low risk, revealing a significant enrichment of M0 and M1 macrophages in the high-risk group. Conversely, resting dendritic cells (DCs) and M2 macrophages were substantially enriched in the low-risk patients (Fig. 9A). Subsequently, the immune status of lung cancer patients was analyzed based on the prognostic model. Stromal, immune, and ESTIMATE scores were calculated for both low- and high-risk patients using the ESTIMATE algorithm. The results showed that low- and high-risk patients had substantially different stromal and Estimate scores (Fig. 9B). Lastly, the immune-infiltrating cells were examined to determine how they correlated with the risk scores. The correlation between activated CD4^+^ T cell memory and risk scores was positive, but the correlation between monocytes, resting mast cells, resting CD4^+^ T cell memory, and resting DCs and the risk score was negative.

### 4.9 Relationship between risk scores and response to immunotherapy

**Fig. 10:**
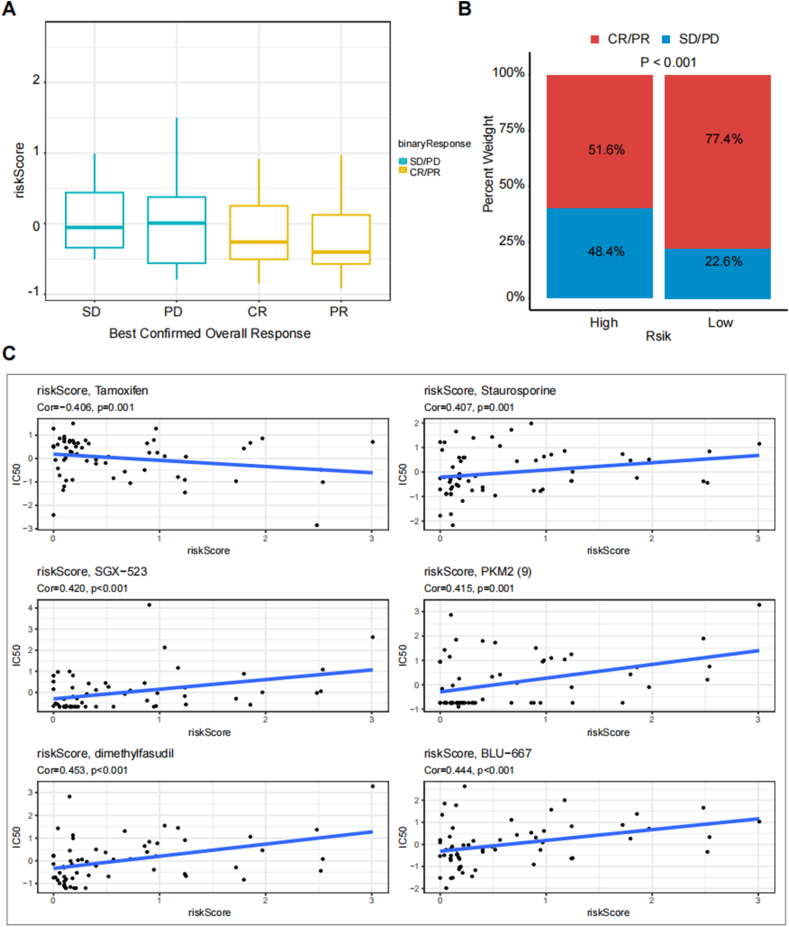
Correlation with Immunotherapy Outcomes and Drug Sensitivity Analysis. (A): The IMvigor210 immunotherapy cohort was utilized to assess the association of risk scores with immunotherapy outcomes. (B): The proportion of patients with high-risk scores achieving CR/PR was considerably smaller than that of those with low-risk scores (p < 0.001). (C): Risk scores exhibited a strong connection with six drugs from the Genomics of Drug Sensitivity in Cancer (GDSC) database.

Finally, we utilized the IMvigor210 immunotherapy cohort to assess the link between risk scores and immunotherapy outcomes. Patients achieving partial response (PR) or complete response (CR) exhibited lower risk scores compared to those with stable disease (SD) or disease progression (PD) (Fig. 10A). Furthermore, in comparison to patients with low-risk scores, a remarkably lower proportion of high-risk patients achieved CR/PR (p < 0.001) (Fig. 10B). Lastly, through Spearman correlation analysis, drugs that were substantially associated with risk scores were identified. The results revealed a substantial link between risk scores and six drugs from the GDSC database (Fig. 10C).

### 4.10 KCNE1, NPC2, and SFTPD are lowly expressed in malignant epithelial cells

**Fig. 11:**
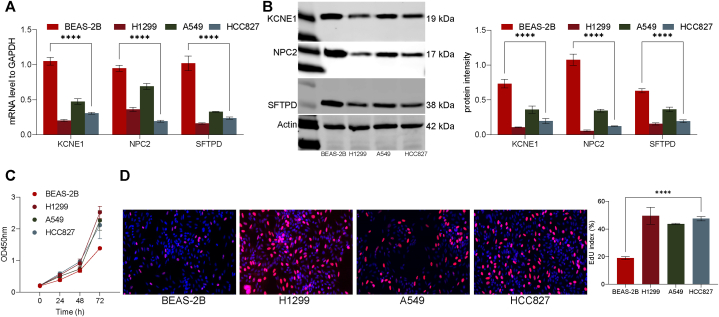
Experimental Validation of Bioinformatics Analysis Results. (A–B): BEAS-2B cells exhibited a remarkable increase in the expression levels of KCNE1, NPC2, and SFTPD in comparison to malignant epithelial cells. (C–D): The BEAS-2B cells exhibited remarkably lower proliferative activity in comparison to the H1299, A549, and HCC827 cells. The experiments were repeated three times, and data were presented as mean ± SD was used to present the data. Significance analysis was conducted using one-way or two-way ANOVA, ANOVA, and post hoc tests using Tukey's multiple comparison test, * * *P < 0.001, * * * *P < 00.0001.

In our previous analysis, 18 genes linked to lysosomes were subjected to differential expression analysis between normal and cancerous epithelial cells. Based on the TCGA database, a prognostic model was established, and the signature composed of three genes (KCNE1, NPC2, SFTPD) was finally selected. Further analysis of the effect of the 3 genes on patient outcomes revealed that all 3 genes were protective factors. To additionally validate the accuracy of the bioinformatics analysis findings, we first examined KCNE1 in normal Epithelial cells BEAS-2B and malignant epithelial cells (lung cancer cell lines, H1299, A549, and HCC827), NPC2, and the mRNA and protein expression of SFTPD. We observed that in the BEAS-2B, the expression levels of KCNE1, NPC2, and SFTPD were significantly higher than in the malignant Epithelial cells (Fig. 11Ã B). Moreover, we further analyzed the malignant proliferation capacity of CCK-8 and EdU, and we discovered that the proliferative activity of H1299, A549, and HCC827 cells was considerably higher than that of BEAS-2B cells (Fig. 11C ∼ D).

### 4.11 Over-expression of KCNE1, NPC2, and SFTPD inhibits the malignant biological behavior of malignant epithelial cells

**Fig. 12:**
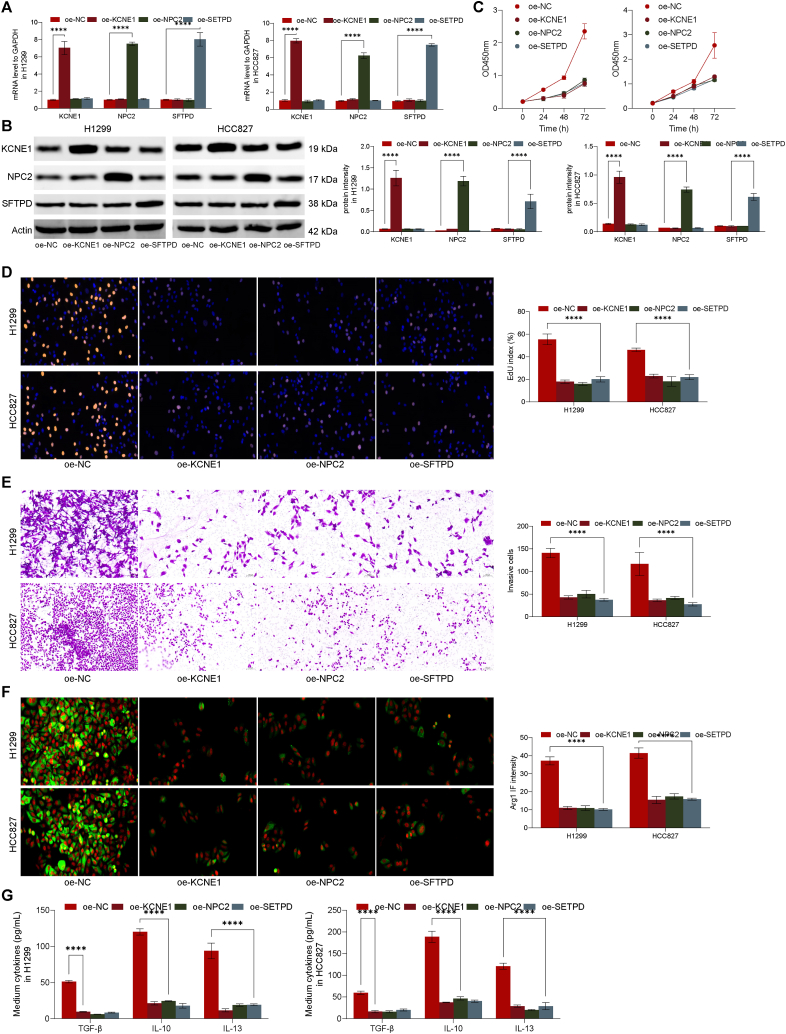
Functional Validation of KCNE1, NPC2, and SFTPD Overexpression in Lung Cancer Cells. (A–B): KCNE1, NPC2, and SFTPD were overexpressed in H1299 and HCC827 cells, and the efficiency of the infection was assessed by qPCR and Western blotting. (C–D): Proliferation ability was analyzed using CCK-8 and EdU staining. (E): The invasive capacity of H1299 and HCC827 cells was assessed using the Transwell assay. (F): H1299 cells were co-cultured with HCC827 cells and M0 macrophages. (G): The amount of tumor-promoting cytokines secreted by cells was significantly reduced after overexpression of KCNE1, NPC2, and SFTPD in HCC827 cells.

To further validate the impacts of KCNE1, NPC2, and SFTPD on malignant epidermal cells in lung cancer, we first overexpressed these genes in H1299 and HCC827 cells and then performed qPCR and WB to determine the efficiency of infection (Fig. 12Ã B). Following that, the proliferative capacity of cells was assessed via CCK-8 and EdU staining. From the results, the expression of KCNE1 in H1299 and the expression of NPC2 and SFTPD in HCC827 cells was significantly impaired (Fig. 12C ∼ D). Moreover, we further used transwell assay to analyze the invasive capacity of H1299 and HCC827 cells, and we found that when H1299 increased KCNE1, NPC2, and SFTPD in HCC827 cells, the invasion ability of cells was significantly inhibited (Fig. 12E). Moreover, we found that patients with low expression of KCNE1, NPC2, and SFTPD had a higher proportion of M2 macrophages. Therefore, to further culture H1299 cells with HCC827 and M0 macrophages, we found that after over-expression of KCNE1, NPC2, SFTPD, M0 macrophage polarization significantly decreased (Fig. 12F), and the amount of tumor-promoting cytokines secreted by cells was significantly reduced (Fig. 12G).

## 5 Discussion

In this study, we found that LSAGs could clearly distinguish normal lung epithelial cells from malignant lung epithelial cells. To accurately predict the prognosis of patients in the TCGA dataset, we developed a three-gene risk model. A correlation analysis was conducted between risk scores and risk grades to ascertain the practical prognostic implications of this model. It was observed that the expression levels of three genes were comparatively lower in high-risk patients as opposed to low-risk patients. The forest plot indicates that HR values for SFTPD, NPC2, and KCNE1 are all less than 1, with NPC2 reaching statistical significance. Subsequently, ROC curves for one, three, and five years were generated, illustrating the model's robust prediction ability. To assess the prognostic model's validity, patients were categorized into subgroups based on clinical characteristics like age, gender, and clinical stage. Comparative analyses were conducted on patients in both high- and low-risk groups. The DCA results demonstrated that the model's predictive performance surpassed that of other clinical indications. This suggests that the model we developed has superior predictive performance in practical scenarios as well.

Increasing research evidence suggests that the onset and progression of cancer are significantly influenced by the TME, which is composed of tumor cells that interact with the surrounding cells through the lymphatic system [[18], [19], [20]]. The significance of investigating the TME in cancer research is underscored by the impact of this interaction on carcinogenesis [21]. Changes in the TME—consisting of immune cells, non-immune cells, and the extracellular matrix—have a substantial impact on the growth of tumors [[22], [23], [24]]. Functional enrichment analysis and immune infiltration analysis were further conducted in this study to assess the possible involvement of LSAG expression in subsets of LUAD patients. Go analysis shows that differentially expressed LSAGs are associated with binding and molecular activity functions, inhibition of DNA damage repair, and tumor growth. KEGG enrichment analysis identified signaling pathways, including IL7 signaling pathway and glycolysis, enriched between high- and low-risk patients.

Another important finding is that, in the LUAD patients, compared the differences of 23 immune cells from high-risk patients and found a significant enrichment of M0 macrophages and M1 macrophages in high-risk patients. Conversely, low-risk patients had a substantial enrichment of M2 macrophages and resting DCs. The differentiation of high-risk and low-risk patients in terms of the infiltration of macrophages suggests that lysosomal-related characteristics may affect the abundance of macrophages in the TME, and consequently the LUAD patients' prognoses. By stimulating the differentiation of tumor-associated macrophages (TAM) and modifying TME, NPC2 may improve the prognosis of human glioblastoma (GBM) [25]. TAM inhibits the invasion of tumor T cells, which in turn promotes the metastasis of LUAD [26]. In vitro, we found that KCNE1, NPC2, and SFTPD are lowly expressed in malignant epithelial, and the proliferation and invasion capacity of cells was inhibited after over-expression. An increased percentage of infiltrated M2 macrophages was discovered in patients who had poor expression of KCNE1, NPC2, and SFTPD. Following the overexpression of KCNE1, NPC2, and SFTPD, M0 macrophages exhibited a substantial reduction in the polarization of M2 macrophages as well as the quantity of tumor-promoting cytokines that were produced by the cells. As the most abundant tumor-infiltrating leukocyte in the TME, TAM can be used as a target for lysosomal-associated DNA nanodevices for tumor remission [27]. A potential novel approach for immune cell-targeted immunotherapy in the future might include targeting TAM at the lysosomal level [28].

Furthermore, the link between immune-infiltrating cells and risk scores was analyzed. The risk score was positively linked to activated CD4^+^ T cell memory, but negatively linked to resting DCs, monocytes, resting mast cells, and resting CD4^+^ T cell memory. Infiltration of T cells is a positive prognostic marker in the majority of solid tumor types, and T cells play a crucial role in tumor immunity [29,30]. Immunotherapies that improve T cell function in tumors are rapidly becoming a standard therapy, with a focus on the recruitment of tumor-infiltrating T cells. Several cytokines exhibiting direct effector roles and the ability to activate other immune cells (including B cells, DCs, and CD8^+^ T cells) are secreted by CD4^+^ T cells [31,32]. The immune response in lung cancer relies heavily on tumor-infiltrating CD4^+^ T cells [33,34]. One way in which CD4^+^ T cells influence tumors is by facilitating the entry of CD8^+^ T cells into the tumor site and the infected mucosa; CD4^+^ T cells are also necessary for the inhibition of angiogenesis at the tumor site [35]. All these findings further suggest that LSAGs influence the prognosis of LUAD patients by affecting tumor immune cell infiltration. However, the relationship between immunomodulatory cells and the expression of LSAGs needs further investigation.

Because the complex TME can cause LUAD cells to become resistant to immune checkpoint inhibitors (ICIs), which can impact the efficacy of immunotherapy, we found that the low-risk and high-risk patients had different expression levels of different immune checkpoint genes. We utilized the IMvigor210 immunotherapy cohort to ascertain the correlation between risk scores and immunotherapy outcomes. Patients who achieved CR or PR showed lower risk scores compared with patients with SD or PD. According to these findings, patients with lower scores could benefit more from these ICIs.

Although previous comparable articles used structural features to predict the prognosis of patients with LUAD, our work still has some strengths. At the cellular level, we further verified the model genes' expression levels and their significance in the TME. However, our study has certain limitations. To further evaluate this trait, future research should use large-scale, multicenter randomized controlled trials. Increasing the number of in vivo and in vitro tests is necessary for future research into the specialized mechanisms of these three genes in lung cancer.

## Data availability statement

The datasets generated and/or analyzed during this study are available from the corresponding authors upon reasonable request.

## Funding

This project has been supported by the National Natural Science Foundation of China (82300068).

## CRediT authorship contribution statement

**Zi-Ming Wang:** Data curation, Writing – original draft. **Zhi-Lin Ning:** Data curation. **Chao Ma:** Formal analysis. **Tang-Bin Liu:** Validation. **Bo Tao:** Writing – review & editing. **Liang Guo:** Writing – review & editing.

## Declaration of competing interest

The authors declare that they have no known competing financial interests or personal relationships that could have appeared to influence the work reported in this paper.
