# Supplementary figures and images for: Low expression of lysosome-related genes KCNE1, NPC2, and SFTPD promote cancer cell proliferation and tumor associated M2 macrophage polarization in lung adenocarcinoma
Source: Heliyon. 2024 Mar 2;10(6):e27575. doi: 10.1016/j.heliyon.2024.e27575 (PMC10950582; doi:10.1016/j.heliyon.2024.e27575)

Fig 11B

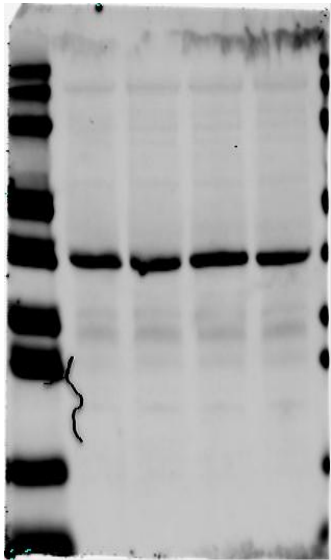

Actin

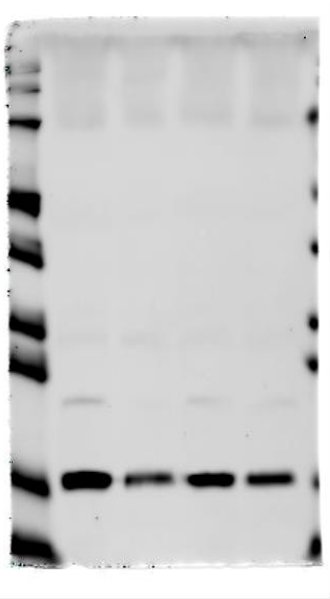

KCNE

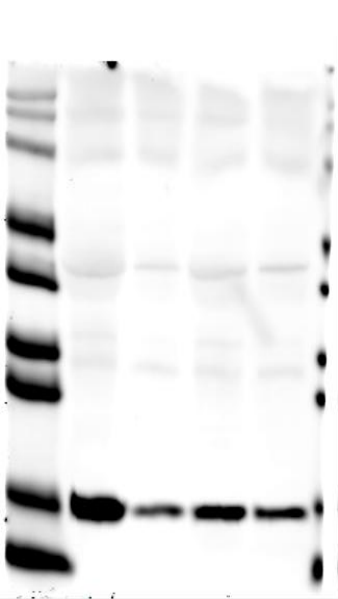

NPC2

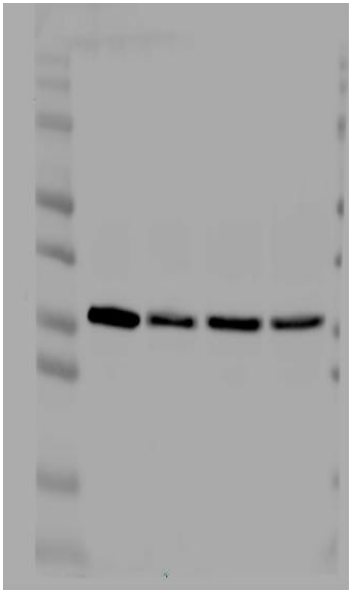

sftpd

Fig 12B

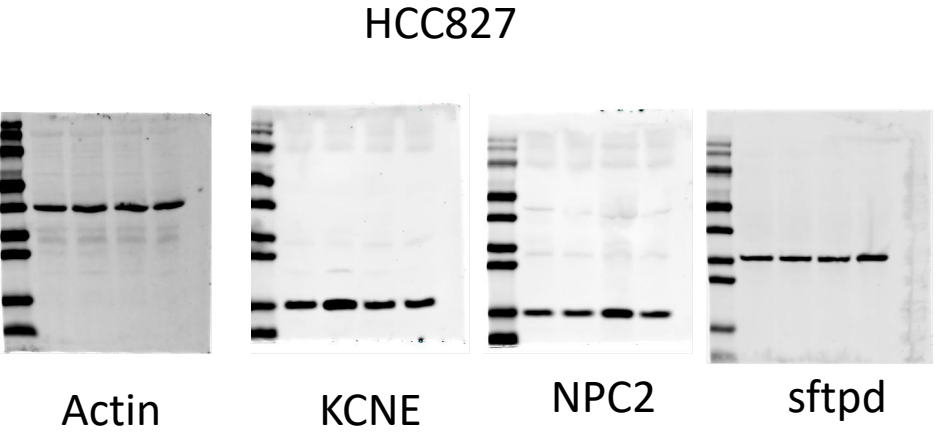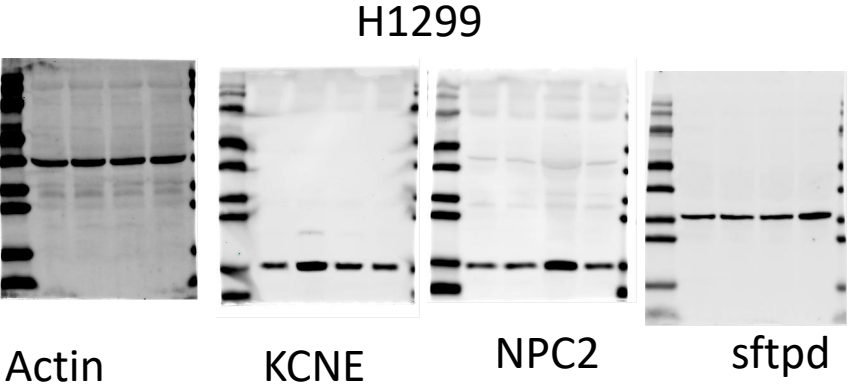

Supplement: Multimedia component 2 [file mmc2.pdf]
